# 

**Published:** 1916-06

**Authors:** 


70th ANNIVERSARY YEAR

### THE DENTAL REGISTER

Edited by Dr NELVILLE S. HOFF
ANN ARBOR, MICH.

VOL. LXX JUNE, 1916 NO. 6

LIBRARY
JUL. 5 - 1916
SURGEON-GENERAL'S OFFICE

THE SAMUEL A. CROCKER CO., Publishers
OHIO DENTAL AND SURGICAL DEPOT
CONRAD BLDG., 18 & 20 W. Seventh St., CINCINNATI, O.

PRICE, ONE DOLLAR A YEAR

Entered at the Post-Office at Cincinnati, O., as second-class mail matter

